# Vitamin D Deficiency Aggravates Hepatic Oxidative Stress and Inflammation during Chronic Alcohol-Induced Liver Injury in Mice

**DOI:** 10.1155/2020/5715893

**Published:** 2020-02-27

**Authors:** Chun-Qiu Hu, Qing-Li Bo, Lan-Lan Chu, Yong-Di Hu, Lin Fu, Guo-Xiu Wang, Yan Lu, Xiao-Jing Liu, Hua Wang, De-Xiang Xu

**Affiliations:** ^1^Department of Toxicology, School of Public Health, Anhui Medical University, Hefei 230032, China; ^2^Department of Nutrition and Hygiene, School of Public Health, Anhui Medical University, Hefei 230032, China; ^3^Department of Respiratory, Second Affiliated Hospital of Anhui Medical University, Hefei 230012, China; ^4^Department of Gastroenterology, Second Affiliated Hospital of Anhui Medical University, Hefei 230012, China; ^5^Department of Paediatrics, First Affiliated Hospital, Anhui Medical University, Hefei 230022, China

## Abstract

Vitamin D deficiency has been reported in alcoholics. This study is aimed at evaluating the effects of vitamin D deficiency on chronic alcohol-induced liver injury in mice. Mice were fed with modified Lieber-DeCarli liquid diets for 6 weeks to establish an animal model of chronic alcohol-induced liver injury. In the VDD+EtOH group, mice were fed with modified diets, in which vitamin D was depleted. Vitamin D deficiency aggravated alcohol-induced liver injury. Furthermore, vitamin D deficiency aggravated hepatocyte apoptosis during alcohol-induced liver injury. Although it has a little effect on hepatic TG content, vitamin D deficiency promoted alcohol-induced hepatic GSH depletion and lipid peroxidation. Further analysis showed that vitamin D deficiency further increased alcohol-induced upregulation of hepatic inducible nitric oxide synthase (*inos*), two NADPH oxidase subunits *p47phox* and *gp91phox*, and heme oxygenase- (HO-) 1. By contrast, vitamin D deficiency attenuated alcohol-induced upregulation of hepatic antioxidant enzyme genes, such as superoxide dismutase (*sod*) 1 and *gshpx*. In addition, vitamin D deficiency significantly elevated alcohol-induced upregulation of hepatic proinflammatory cytokines and chemokines. Taken together, these results suggest that vitamin D deficiency aggravates hepatic oxidative stress and inflammation during chronic alcohol-induced liver injury.

## 1. Introduction

Alcohol abuse is a serious public health concern responsible for a major cause of morbidity and mortality worldwide [1]. Chronic heavy drinking results in alcoholic liver disease (ALD), characterized by a varied spectrum of liver injury ranging from simple steatosis to alcoholic hepatitis, necrosis, progressive fibrosis, and even hepatocellular carcinoma [2]. Worldwidely, approximately 3.3 million people die each year from alcohol consumption, which accounts for approximately 5.9% of all deaths [3]. The liver is the main site of alcohol metabolism, where about 90% alcohol is metabolized into acetaldehyde by alcohol dehydrogenase (ADH), and subsequently metabolized into acetic acid by aldehyde dehydrogenase (ALDH) [4]. Excessive alcohol and its metabolites activate cytochrome P450 2E1 (CYP2E1) resulting in excess production of reactive oxygen species (ROS) [5]. Excess ROS induces depletion of antioxidants, hepatic oxidative stress, and damage to DNA, proteins, and lipids [6].

Vitamin D is essential for the maintenance of calcium homeostasis and bone metabolism [7]. Recently, vitamin D is well-known for its nonclassical function including the regulation of immune response and anticancer activity [8, 9]. Vitamin D deficiency, defined as lower than 50 nmol/L of 25-hydroxy vitamin D (25(OH)D), is increasingly recognized as a global public health problem [10]. Indeed, vitamin D deficiency is prevalent in alcoholics [11]. A clinical study found that low 25(OH)D levels were associated with increased mortality in ALD [12]. Another prospective study found that supplementation with vitamin D reduced the Child-Pugh score in patients with alcoholic liver cirrhosis [13]. Increasing data demonstrate that vitamin D has an antioxidant activity [14–16]. Several studies have considered its antioxidant potential to be even stronger than vitamin E and melatonin [17]. According to a recent report, vitamin D insufficiency aggravated hepatic inflammation and oxidative stress in patients with HCV [18]. However, it remains unclear whether vitamin D deficiency aggravates hepatic oxidative stress and inflammation in the process of chronic alcohol-induced liver injury.

In the present study, a chronic alcoholism model was established using Lieber-DeCarli diet containing alcohol feeding in mice for 4 weeks. The effects of vitamin D deficiency on chronic alcoholic liver injury were assessed, and the underlying molecular mechanisms were also evaluated in terms of alcohol metabolism, lipid, inflammation, and oxidative stress. The activities of hepatic ADH and ALDH, hepatic triglyceride content, and lipid accumulation; the expression of proinflammatory and chemokine genes; the oxidative stress parameters including the levels of glutathione (GSH), glutathione disulfide (GSSG), and malondialdehyde (MDA); and the expression of inducible nitric oxide synthase (INOS), NADPH oxidase subunits, antioxidant enzyme genes, and heme oxygenase- (HO-) 1 were measured.

## 2. Material and Methods

### 2.1. Chemicals and Reagents

Absolute ethanol (99.9%) was purchased from Sigma Chemical Co. (St. Louis, MO, USA). 125 I-based T radioimmunoassay (RIA) kit was purchased from DiaSorin (DiaSorin Inc., Stillwater, MN, USA). HO-1 and *β*-actin antibodies were from Cell Signaling Technology (Beverly, MA). Chemiluminescence (ECL) detection kit was from Pierce Biotechnology (Rockford, IL, USA). TRI reagent was from Invitrogen (Carlsbad, CA, USA). RNase-free DNase was from Promega Corporation (Madison, WI, USA). All other reagents were purchased from Sigma Chemical Co. or as indicated in the specified methods.

### 2.2. Animals and Treatments

Six-week-old SPF C57BL/6J mice were purchased from Beijing Vital River, whose foundation colonies were all introduced from Charles River Laboratories, Inc. (Wilmington, MA, USA). The animals were allowed free access to food and water at all times and were maintained on a 12-hour light/dark cycle in a controlled temperature (20-25°C) and humidity (50 ± 5%) environment for one week. Mice were randomly assigned into four groups (10 animals each group). In the Ctrl group, mice were fed with a control liquid diet (containing 1000 IU VitD_3_/kg, code: TP4030C). In the EtOH group, mice were fed with containing 4% (*w*/*v*) alcohol liquid diet (containing 1000 IU VitD_3_/kg, code: TP4030B). In the VDD group, mice were fed with the control liquid diet, in which vitamin D was depleted (lower than 25 IU VitD_3_/kg). In the VDD+EtOH group, mice were fed with alcohol liquid diet, in which vitamin D was depleted (lower than 25 IU VitD_3_/kg). The liquid diet (Trophic Animal Feed High-Tech Co. Ltd., Nantong, Jiangsu, China) provides 1 kcal/mL based on the Lieber-DeCarli formulation, and 35% of calories are provided from fat, 19% from carbohydrate, 18% from protein, and 28% from isocaloric maltose dextrin (control liquid diet) or alcohol (4% (*w*/*v*) alcohol-containing liquid diet). The diets were freshly prepared from powder and provided daily at 5:00 p.m. Mice were fed the Lieber-DeCarli control diet for one week for adaptation to liquid diet and then fed a different proportion of Lieber-DeCarli alcohol diet for one week starting at a ratio of the Lieber-DeCarli control diet to the Lieber-DeCarli alcohol diet of 2 : 1 to 1 : 1 and increasing to 1 : 2 on days 2, 4, and 6, with the exception of mice in the normal control group. At the end of the adaptation period, mice were fed a complete Lieber-DeCarli alcohol diet. The Ctrl group was pair-fed with the EtOH group, and the other groups were fed ad libitum. Mice were housed under the ultraviolet section of light (290-315 nm) which was filtered, and they were inspected daily for food intake and weighted weekly. After 6 weeks on the diet intervention, the mice were euthanized. Blood samples were collected for measurement of 25(OH)D and biochemical parameters. The liver was collected and either frozen immediately in liquid nitrogen for subsequent experiments, or fixed in 4% paraformaldehyde for histology, or frozen-fixed in OCT mounting media for Oil Red O staining. This study was approved by the Association of Laboratory Animal Sciences and the Center for Laboratory Animal Sciences at Anhui Medical University (permit number: 17-0014). All procedures on animals followed the Guide for the Care and Use of Laboratory Animals published by the US National Institutes of Health (NIH Publication No. 85-23, revised 1996).

### 2.3. Measurement of 25(OH)D

The radioimmunoassay (RIA) kit with 125 I-labeled 25(OH)D as a tracer (DiaSorin Inc., Stillwater, MN, USA) was used to measure the concentration of serum 25 (OH)D [19]. Serum 25(OH)D is expressed as ng/mL.

### 2.4. Serum Biochemical Parameters

Serum triglyceride (TG), total cholesterol (CHOL), high-density lipoprotein cholesterol (Chol-HDL), low-density lipoprotein cholesterol (Chol-LDL), very low-density lipoprotein cholesterol (Chol-VLDL), alanine transaminase (ALT), and aspartate aminotransferase (AST) were determined by routine laboratory methods using an autoanalyzer (Roche, Modular DPP, NO.1549-06).

### 2.5. Histological Examination

Formalin-fixed liver sections were stained with hematoxylin and eosin (H&E) and then observed using an inverted microscope (Olympus IX-73, Olympus) for pathological score. Hepatic pathological scores were independently evaluated by three pathologists in twelve randomly fields from each slide at a magnification of ×200. The pathological scoring standard was referred to our previous research [20].

### 2.6. Terminal dUTP Nick-End Labeling (TUNEL) Staining

For the detection of apoptosis, paraffin-embedded sections were stained with the TUNEL technique using an in situ apoptosis detection kit (Promega Madison, WI) according to the manufacturer's protocols. TUNEL-positive cells were counted in eight randomly selected fields from each slide at a magnification of ×200.

### 2.7. ADH and ALDH Activities

Hepatic ADH and ALDH activities in the liver were determined by kits (Nanjing Jiancheng Bioengineering Institute, Nanjing, China) according to the manufacturer's instructions. Liver tissues were homogenized in ice-cold isotonic saline. The homogenate was centrifuged at 2500 g for 10 min at 4°C to obtain supernatant, which was collected for the measurement of hepatic ADH. Each sample of 50 *μ*L was mixed with 150 *μ*L of detection reagent (a mixture containing NAD+ and ethanol in buffer according to the instructions of the manufacturer) and monitors the conversion of NAD+ to NADH by measuring the changes in absorbance at 340 nm for 10 min after the initiation of the enzyme reaction. For hepatic ALDH assay, the liver tissue was homogenized in extraction reagent. The homogenate was centrifuged at 1000 g for 20 min at 4°C to obtain supernatant, which was collected for the measurement. Each sample of 200 *μ*L was mixed with 800 *μ*L of detection reagent and monitored the conversion of NAD+ to NADH by measuring the changes in absorbance at 340 nm for 60 sec after the initiation of the enzyme reaction.

### 2.8. Hepatic TG Measurement

Hepatic TG was extracted using the method developed by Bligh and Dyer with minor modification [21]. Liver samples were homogenized in ice-cold 2x phosphate-buffered saline (PBS). Triglyceride was extracted with methanol/chloroform (1 : 2), dried, and resuspended in 5% fat-free bovine serum albumin. The level of triglyceride was determined using a commercially available kit (Zhejiang Dongou Diagnostics Co., Ltd, Wenzhou, China) according to the manufacturer's protocol. Hepatic triglyceride content was expressed as *μ*mol/g liver.

### 2.9. Oil Red O Staining

Frozen sections (10 *μ*m) were stained with Oil Red O for 10 min, washed, and counterstained with hematoxylin for 45 s. Representative photomicrographs were captured at 200x magnification using a system incorporated in the microscope.

### 2.10. Glutathione (GSH) and Glutathione Disulfide (GSSG) Measurement

Liver samples were homogenized in 5% trichloroacetic acid, then centrifuged at 3500 rpm for 10 min. The supernatant was used to detect liver GSH and GSSG level by an assay kit (Nanjing Jiancheng Bioengineering Institute, Nanjing, China) according to the manufacturer's guidelines.

### 2.11. Lipid Peroxidation Assay

Lipid peroxidation was quantified by measuring malondialdehyde (MDA) as described previously. Liver tissue was homogenized in 9 volumes of 50 mm Tris-HCl buffer (pH 7.4) containing 180 mm KCl, 10 mm EDTA, and 0.02% butylated hydroxytoluene. To 0.2 mL of the tissue homogenate, 0.2 mL of 8.1% sodium dodecylsulfate, 1.5 mL of 20% acetic acid, 1.5 mL of 0.9% thiobarbituric acid, and 0.6 mL of distilled water were added and vortexed. The reaction mixture was placed in a water bath at 95°C for 40 min. After cooling on ice, 1.0 mL of distilled water and 5.0 mL of butanol/pyridine mixture (15 : 1, *v*/*v*) were added and vortexed. After the reaction mixture was centrifuged at 10,000 g for 10 min, absorbance of the supernatants was determined at 532 nm.

### 2.12. Isolation of Total RNA and Real-Time RT-PCR

Total RNA was extracted from liver tissues using TRI reagent. Total RNA (1.0 *μ*g) was treated with RNase-free DNase and reverse-transcribed with AMV (Promega Corp., Madison, WI, USA). Real-time RT-PCR was performed with a LightCycler 480 SYBR Green I Kit (Roche Diagnostics GmbH, Manheim, Germany) using gene-specific primers as listed in Table 1. 18s was used as an endogenous control to normalize the expression of the selected genes. The amplification reactions were carried out on a LightCycler 480 Instrument (Roche Diagnostics GmbH, Mannheim, Germany) with an initial hold step (95°C for 5 min) and 50 cycles of a three-step PCR (95°C for 15 sec, 60°C for 15 sec, and 72°C for 30 sec). The relative ratio of the target gene was calculated using a LightCycler 480SYBR Green I Kit software (Roche Diagnostics) (Roche, version 1.5.0).

### 2.13. Immunoblots

For total protein extraction, the methods were used on the basis of our previous study [22]. 50 mg liver tissue was used to fabricate lysate in 300 mL RIPA buffer (50 mM Tris-HCl, pH 7.4, 150 mM NaCl, 1 mM EDTA, 1% Triton X-100, 1% sodium deoxycholate, 0.1% sodium dodecylsylphate, and 1 mM phenylmethylsulfonyl fluoride) supplemented with a cocktail of protease inhibitors. The concentration of protein was determined by the bicinchoninic acid (BCA) protein assay. For immunoblots, the same amount of protein was separated electrophoretically by SDS-PAGE and transferred to a polyvinylidene fluoride membrane. The membranes were incubated for 2 h with HO-1 antibody; *β*-actin was used as a loading control for total proteins. After washes in DPBS containing 0.05% Tween-20 four times for 10 min each, the membranes were incubated with goat anti-rabbit IgG antibody for 2 h. The membranes were then washed for four times in DPBS containing 0.05% Tween-20 for 10 min each, followed by signal development using an ECL detection kit. Relative quantification of each protein was calculated after normalization to loading control protein by densitometric analysis with Image-Pro Plus V7 software.

### 2.14. Statistical Analysis

Normally distributed data were expressed as means ± SEM. A comparison between two groups (Ctrl versus EtOH, Ctrl versus VDD, and EtOH versus VDD+EtOH) was performed using a *t* test. ANOVA and the Student-Newman-Keuls post hoc test were used to determine differences among different groups. *P* < 0.05 was considered statistically significant.

## 3. Results

### 3.1. Body Weight Growth and Caloric Intakes

**Figure 1:**
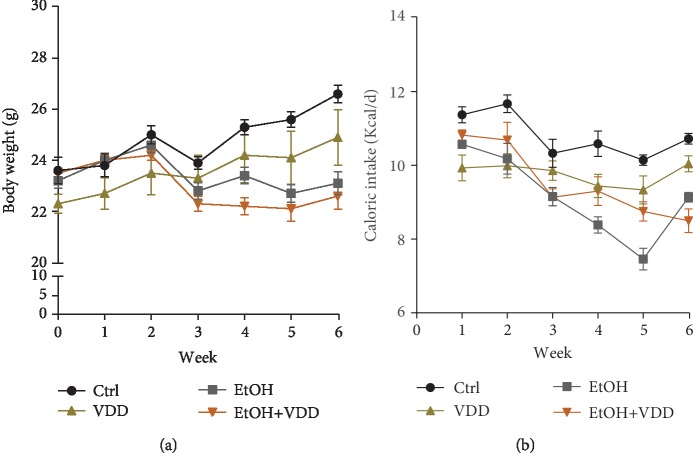
Effects of vitamin D deficiency on body weight growth and caloric intake. In the Ctrl group, mice were fed with a control liquid diet. In the EtOH group, mice were fed with containing 4% (*w*/*v*) alcohol liquid diet. In the VDD group, mice were fed with the control liquid diet, in which vitamin D was depleted. In the VDD+EtOH group, mice were fed with alcohol liquid diet, in which vitamin D was depleted. Mice were inspected daily for food intake and weighted weekly for body weight. (a) Body weight. (b) Caloric intake. All data were expressed as means ± SEM (*n* = 10).

As shown in Figure 1(a), the body weight of the alcohol group showed a significant decrease as compared with the controls, whereas no difference was observed between VDD-fed mice and controls. Additionally, there was no significant difference in body weight between the EtOH group and the EtOH+VDD group over 6-week diet intervention, indicating that vitamin D deficiency did not affect weight gain. The effects of vitamin D deficiency on caloric intakes are analyzed in Figure 1(b). The energy intake of the EtOH group was significantly lower than that of controls, while there was no difference in energy intake between the EtOH group and the EtOH+VDD group, indicating that vitamin D deficiency had no significant influence on energy intake, which was consistent with the change of body weight.

### 3.2. Serum 25(OH)D Concentration

**Figure 2:**
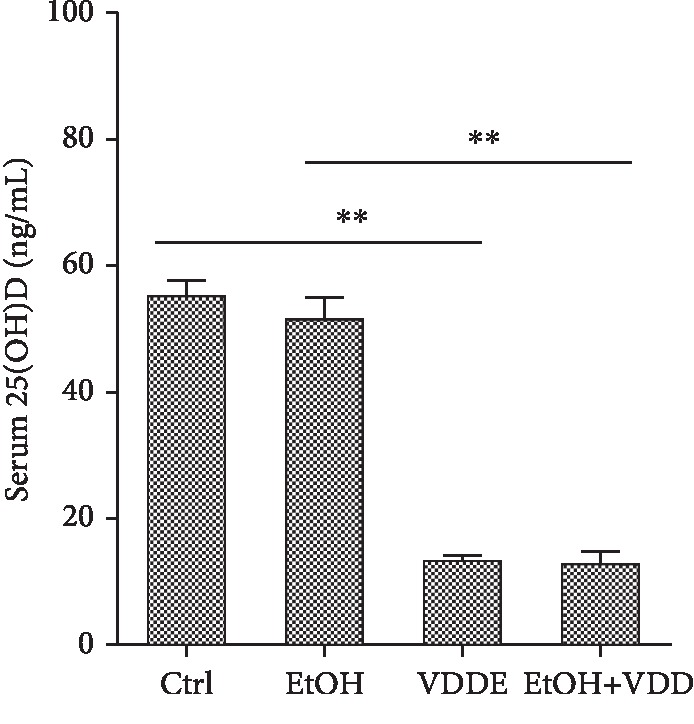
Effects of vitamin D deficiency on serum 25(OH)D concentration. In the Ctrl group, mice were fed with a control liquid diet. In the EtOH group, mice were fed with containing 4% (*w*/*v*) alcohol liquid diet. In the VDD group, mice were fed with the control liquid diet, in which vitamin D was depleted. In the VDD+EtOH group, mice were fed with alcohol liquid diet, in which vitamin D was depleted. All mice were euthanized after 6-week diet intervention. Serum samples were collected. Serum 25(OH)D was measured using 25(OH)D RIA kits. All data were expressed as means ± SEM (*n* = 10). ^∗∗^*P* < 0.01.

The effects of vitamin D deficiency diet on serum 25(OH)D concentration were analyzed. As shown in Figure 2, serum 25(OH)D concentration was reduced to 13.32 ± 0.81 ng/mL in the VDD group and 12.78 ± 2.1 ng/mL in the VDD+EtOH group, significantly lower than those in the control (55.34 ± 2.59 ng/mL) and the EtOH (51.68 ± 3.53 ng/mL) groups.

### 3.3. Vitamin D Deficiency Does Not Affect ADH and ALDH Activities in the Liver

The effects of vitamin D deficiency on ADH and ALDH activities in the liver were analyzed. As shown in Table 2, feeding of alcohol induced marked change of ADH activity in the EtOH group and the VDD+EtOH group, whereas no significant difference was observed between the VDD group and the Ctrl group or the EtOH group and the VDD+EtOH group. A similar tendency was also shown for ALDH activity among different groups. These data indicated that vitamin D deficiency does not affect the activities of ADH and ALDH in the liver.

### 3.4. Vitamin D Deficiency Does Not Aggravate Alcohol-Induced TG Elevation and Hepatic Lipid Accumulation

**Figure 3:**
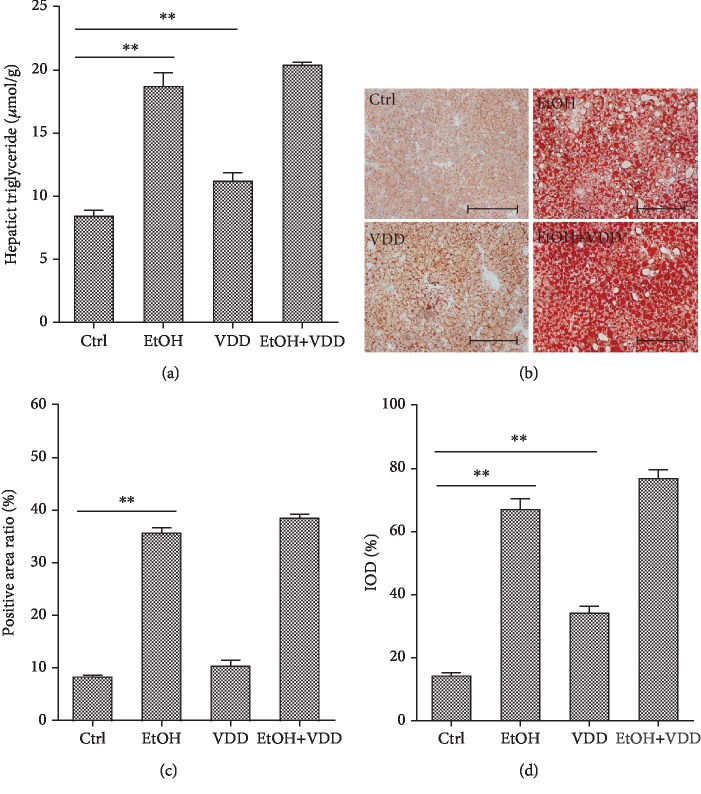
Effects of vitamin D deficiency on alcohol-induced hepatic triglyceride elevation and hepatic lipid accumulation. In the Ctrl group, mice were fed with a control liquid diet. In the EtOH group, mice were fed with containing 4% (*w*/*v*) alcohol liquid diet. In the VDD group, mice were fed with the control liquid diet, in which vitamin D was depleted. In the VDD+EtOH group, mice were fed with alcohol liquid diet, in which vitamin D was depleted. All mice were euthanized after 6-week diet intervention. Liver samples were collected. (a) Hepatic triglyceride content. (b) Liver sections were stained with Oil Red O. Original magnification: 200x. (c) Quantitative analysis was performed using a positive area ratio. (d) Quantitative analysis was performed using integral optical density (IOD). All data were expressed as means ± SEM (*n* = 10). ^∗∗^*P* < 0.01.

The effects of vitamin D deficiency on hepatic TG content and hepatic lipid accumulation are analyzed in Figure 3. As expected, feeding of alcohol significantly elevated hepatic TG content (Figure 3(a)). Correspondingly, an obvious hepatic lipid accumulation, as determined by Oil Red O staining, was observed in alcohol-fed mice (Figures 3(b)–3(d)). A similar tendency was also found between VDD-fed mice and controls. However, vitamin D deficiency had a little effect on alcohol-induced elevation of both hepatic TG content and lipid accumulation. Next, the effects of vitamin D deficiency on serum lipid were analyzed. As shown in Table 3, a similar phenomenon was observed on the level of serum TG, whereas no difference was observed in the level of CHOL, Chol-HDL, Chol-LDL, and Chol-VLDL among different groups.

### 3.5. Vitamin D Deficiency Exacerbates Alcohol-Induced Liver Injury

**Figure 4:**
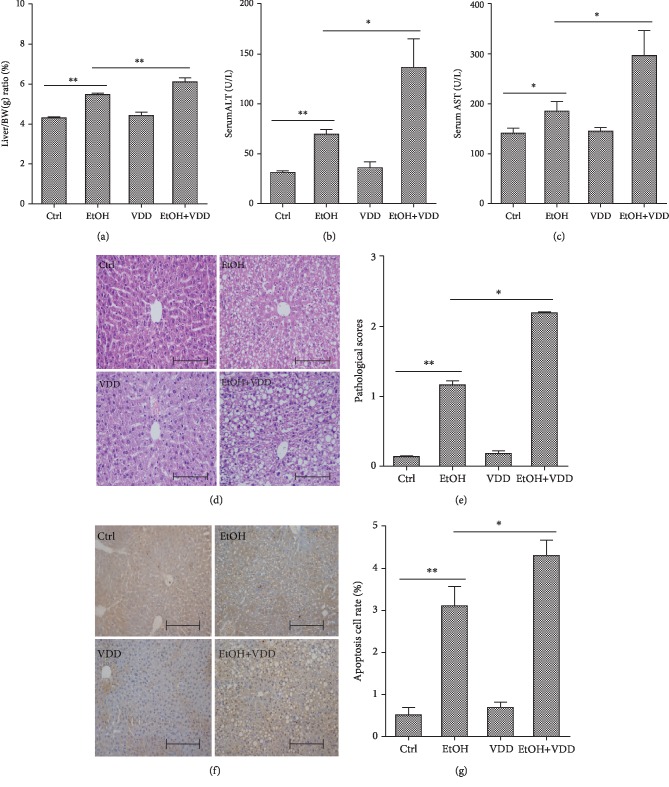
Effects of vitamin D deficiency on alcohol-induced liver injury. In the Ctrl group, mice were fed with a control liquid diet. In the EtOH group, mice were fed with containing 4% (*w*/*v*) alcohol liquid diet. In the VDD group, mice were fed with the control liquid diet, in which vitamin D was depleted. In the VDD+EtOH group, mice were fed with alcohol liquid diet, in which vitamin D was depleted. All mice were euthanized after 6-week diet intervention. Serum and liver samples were collected. (a) Relative liver weight. (b) Serum ALT activity. (c) Serum AST activity. (d) Liver sections were stained with H & E. Original magnification: 200x. (e) Pathological scores were evaluated. (f) Hepatocyte apoptosis was determined using TUNEL assay. Original magnification: 200x. (g) TUNEL+hepatocytes were evaluated. All data were expressed as means ± SEM (*n* = 10). ^∗^*P* < 0.05, ^∗∗^*P* < 0.01.

The liver to body weight ratio was compared among four groups and is shown in Figure 4(a). As expected, the liver index was significantly increased in the EtOH group, whereas no difference was observed between the VDD group and the Ctrl group. Interestingly, vitamin D deficiency aggravated alcohol-induced elevation of liver index. The effects of vitamin D deficiency on liver function during alcohol-induced liver injury are analyzed in Figures 4(b) and 4(c). As expected, long-term alcohol consumption significantly increased the activities of serum ALT and AST, and these elevations were further increased when vitamin D in feed was depleted. Vitamin D deficiency alone did not affect the activities of serum ALT and AST. Histology showed a normal lobular architecture and cell structure in the livers of the Ctrl and the VDD groups, but extensive portal inflammation, hemorrhagic necrosis, and increased inflammatory cell infiltration were observed in the liver of the EtOH and the EtOH+VDD groups (Figures 4(d) and 4(e)). Hepatocyte apoptosis was detected using TUNEL staining. As shown in Figures 4(f) and 4(g), numerous TUNEL-positive hepatocytes were observed in liver tissues obtained from mice in the EtOH group and the EtOH+VDD group. However, few TUNEL-positive hepatocytes were observed in the liver of mice in the Ctrl group and the VDD group. Further analysis showed that the number of TUNEL-positive hepatocytes in the EtOH+VDD group was more than that of the EtOH group.

### 3.6. Vitamin D Deficiency Aggravates Alcohol-Induced Upregulation of Hepatic Proinflammatory Cytokines and Chemokines

**Figure 5:**
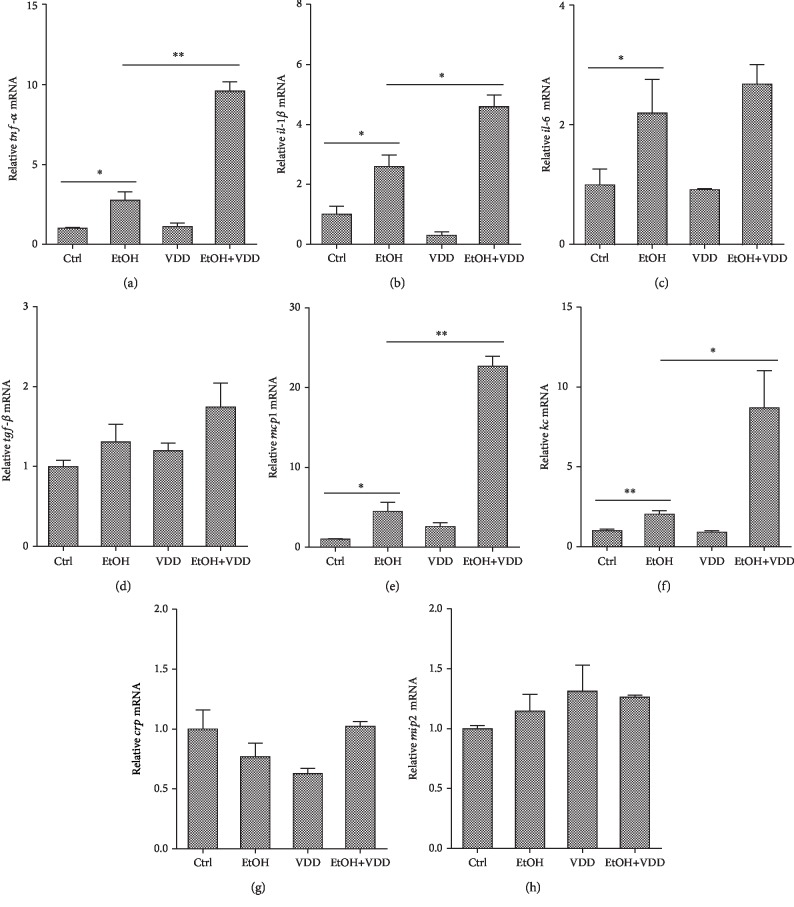
Effects of vitamin D deficiency on alcohol-induced hepatic proinflammatory cytokines and chemokines. All mice were euthanized after 6-week diet intervention. Liver samples were collected. (a–d) Hepatic proinflammatory cytokines mRNA was measured using real-time RT-PCR. (a) tnf-*α*; (b) il-1*β*; (c) il-6; (d) tgf-*β*. (e–h) Hepatic chemokine mRNA was measured using real-time RT-PCR. (e) mcp-1; (f) kc; (g) crp; (h) mip2. All experiments were duplicated for three times. Data were expressed as means ± SEM (*n* = 10). ^∗^*P* < 0.05, ^∗∗^*P* < 0.01.

The effects of vitamin D deficiency on alcohol-induced hepatic proinflammatory and chemokine genes were analyzed. As shown in Figures 5(a)–5(d), mRNA levels of hepatic *tnf-α*, *il-1β*, and *il-6*, three proinflammatory genes, were significantly increased. Vitamin D deficiency significantly elevated alcohol-induced upregulation of hepatic *tnf-α* and *il-1β* mRNAs. As shown in Figures 5(e)–5(h), mRNA levels of hepatic keratinocyte chemoattractant (*kc*) and monocyte chemotactic protein- (*mcp*-) 1, two chemokine genes, were markedly upregulated by alcohol feeding. Interestingly, vitamin D deficiency significantly elevated alcohol-induced upregulation of hepatic chemokines. On the other hand, there is no difference that was observed in *tgf-β*, *crp*, and *mip2* mRNAs among different groups. In addition, vitamin D deficiency alone did not affect the expression of these proinflammatory and chemokine genes.

### 3.7. Vitamin D Deficiency Exacerbated Alcohol-Induced Liver Oxidative Stress

**Figure 6:**
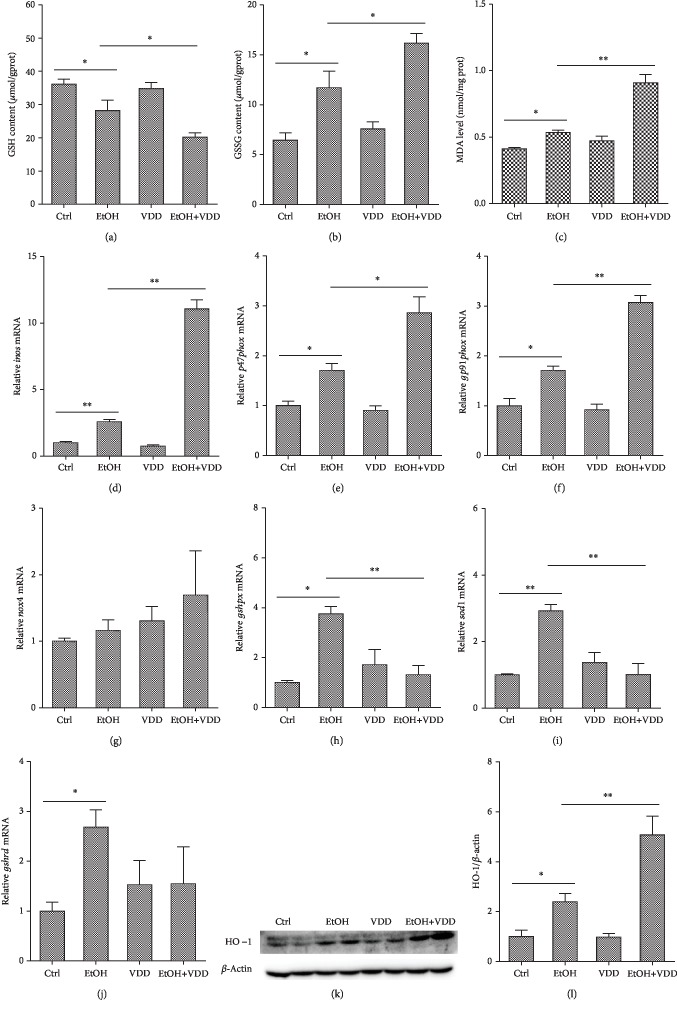
Effects of vitamin D deficiency on alcohol-induced hepatic oxidative stress. All mice were euthanized after 6-week diet intervention. Liver samples were collected. (a) Hepatic GSH content. (b) Hepatic GSSG content. (c) Hepatic MDA content. (d) Hepatic inos mRNA was measured using real-time RT-PCR. (e–g) Hepatic NADPH oxidase subunit mRNAs were measured using real-time RT-PCR. (e) p47phox; (f) gp91phox; (g) nox4. (h–j) Hepatic antioxidase mRNA was measured using real-time RT-PCR. (h) gshpx; (i) sod1; (j) gshrd. (k) Hepatic HO-1 was measured using Western blot. (l) HO-1 were normalized to the level of *β*-actin in the same sample. All experiments were duplicated for three times. Data were expressed as means ± SEM (*n* = 10). ^∗^*P* < 0.05, ^∗∗^*P* < 0.01.

The effect of vitamin D deficiency on alcohol-induced hepatic GSH depletion and lipid peroxidation was investigated. As shown in Figure 6, no significant difference on hepatic GSH, GSSG, and MDA was observed between VDD-fed mice and controls. As expected, alcohol feeding resulted in an obvious decrease in hepatic GSH and the increases in GSSG and MDA levels. Interestingly, vitamin D deficiency aggravated alcohol-induced hepatic GSH depletion and GSSG and MDA elevation (Figures 6(a) and 6(c)). The effect of vitamin D deficiency on alcohol-induced hepatic *inos* mRNA upregulation is presented in Figure 6(d). As expected, hepatic *inos* mRNA was obviously upregulated in alcoholic mice. Of interest, vitamin D deficiency aggravated alcohol-induced upregulation of *inos* mRNA in the liver. In addition, vitamin D deficiency alone did not affect the level of hepatic *inos* mRNA.

The effects of vitamin D deficiency on the expression of hepatic NADPH oxidases were analyzed. As shown in Figures 6(e)–6(g), *p47phox* and *gp91phox*, two NADPH oxidase subunits, were upregulated in alcohol-fed mice. Interestingly, alcohol-induced elevation of hepatic *p47phox* and *gp91phox* was further aggravated when vitamin D in feed was depleted. No significant difference in the expression of hepatic *nox4* was observed among different groups.

The effect of vitamin D deficiency on the expression of hepatic antioxidant enzymes is presented in Figures 6(h)–6(j). As seen, the mRNA level of hepatic *gshpx*, *sod1*, and *gshrd* was upregulated in the alcohol-fed mice. Of interest, vitamin D deficiency attenuated alcohol-induced upregulation of *gshpx* and *sod1*. Vitamin D deficiency alone had a little effect on hepatic antioxidant enzyme genes.

Protein expression of HO-1, a marker of oxidative stress, was examined. As shown in Figures 6(k) and 6(l), hepatic HO-1 was upregulated in the EtOH group. Vitamin D deficiency aggravated alcohol-induced upregulation of hepatic HO-1. In addition, vitamin D deficiency alone had a little effect on the protein levels of hepatic HO-1.

## 4. Discussion

In the present study, the effects of vitamin D deficiency on chronic alcohol-induced liver injury were investigated in a mouse model. Our data showed that vitamin D deficiency exacerbated alcohol-induced liver pathological damage and dysfunction. In addition, vitamin D deficiency aggravated liver cell apoptosis during alcohol-induced liver injury. Meanwhile, we evaluated the underlying mechanisms of the aggravating effect of vitamin D deficiency by examining hepatic alcohol metabolism, lipid, inflammation, and oxidative stress. Our results suggest that vitamin D deficiency which aggravates chronic alcohol-induced liver injury is associated with promoting hepatic inflammation and oxidative stress.

The liver is the most important organ for metabolizing alcohol, and most of alcohol ingested is metabolized by hepatic metabolic enzymes. ADH and ALDH are considered to be essential for the metabolism of alcohol. In this study, our results showed that the activities of ADH and ALDH were increased after alcohol feeding in the mice, which should be an adaptive response to alcohol stimulation. Vitamin D deficiency had no effect on ADH and ALDH activities. In contrast, a recent study reported that vitamin D attenuated alcohol-induced HepG2 cell injury partially by upregulating ALDH2 expression [23]. One possible explanation for this discrepancy could be that in our study, we only focus our attention on the activity of ALDH. In addition, the discrepancy between experiments may be related to the study subjects. However, we do not completely exclude the possibility that vitamin D deficiency which aggravates alcohol-induced liver injury is related to alcohol metabolism. Thus, further studies will need to confirm this assertion.

Along with alcohol metabolism, reactive oxygen species (ROS) were simultaneously generated. The generation and accumulation of ROS exacerbate hepatic oxidative stress, which is known to be a key factor in the pathogenesis of alcohol-induced liver injury [24]. NO is representative of ROS, which was induced by iNOS. NO interacts with superoxide anion radicals and forms ONOO-, a highly destructive oxidizing agent that induces protein nitration. Meanwhile, MDA is the main product of lipid peroxidation induced by ROS and is commonly used as an index for the evaluation of radical-mediated oxidative stress. Naturally, the body possesses antioxidant defense systems to protect themselves against ROS-induced oxidative effects. GSH is the most abundant thiol in mammals that scavenges ROS from the body and is of great importance in the hepatic antioxidant system. Furthermore, GSH can be oxidized to GSSG, which in turn can revert to GSH, regulating redox homeostasis. In the present study, chronic alcohol feeding increased hepatic *inos* mRNA, MDA, and GSSG contents and decreased GSH content. Interestingly, vitamin D deficiency aggravated alcohol-induced elevation of *inos* mRNA, MDA, and GSSG contents and reduction of GSH content. These results indicating alcohol-induced ROS accumulation increased lipid peroxidation and exacerbated hepatic oxidative stress. Vitamin D deficiency may recruit free radicals and promote lipid peroxidation through attenuating the antioxidant defense system in alcohol-induced liver injury. Consistent with our research, antioxidant capacity of vitamin D has been found in in vivo and in vitro experiments [25, 26]. An earlier study showed that vitamin D may have a protective role against hepatic oxidative stress [17]. A recent study demonstrated that 25(OH)D deficiency increased liver injury in patients with hepatitis C virus, in part due to increased oxidative stress [18]. However, the mechanism through which vitamin D deficiency promotes oxidative stress remains obscure. Several reports indicated that the protective effect of vitamin D3 against oxidative stress was associated with the regulation of NADPH oxidases and antioxidant enzymes [15, 16, 25, 27]. Our previous study also found that vitamin D3 pretreatment alleviates LPS-induced real oxidative stress through regulating oxidant and antioxidant enzyme genes [28]. NADPH oxidases, which are composed of several membrane-associated subunits including p22phox, p47phox, p67phox, and gp91phox, are important enzymatic sources of cellular ROS in the pathogenesis of alcohol-induced liver injury [29–31]. At the same time, the antioxidant enzymes, including sod1, gshpx, and gshrd, represent the defense response to excessive free radicals. The effects of alcohol exposure on the activity or content of antioxidant enzymes are rather controversial in the literature, depending on the model, diet, amount, and time of alcohol feeding [32–36]. The present study found that the expression of hepatic *p47phox*, *gp91phox*, and *nox4*, three NADPH oxidase subunits, was upregulated during alcohol-induced liver injury. Meanwhile, the hepatic *sod1*, *gshpx*, and *gshrd* mRNAs were also upregulated by alcohol feeding, suggesting a protective role of these enzymes against alcohol-induced oxidative stress. Interestingly, vitamin D deficiency further increased alcohol-induced upregulation of hepatic NADPH oxidase subunits and significantly attenuated alcohol-induced upregulation of hepatic antioxidant enzymes *gshpx* and *sod1*. HO-1, an inducible form of the rate-limiting enzyme that catabolizes free heme, could regulate the content of downstream antioxidant enzymes and inhibit inflammatory response. In this study, the protein level of hepatic HO-1 was upregulated by alcohol feeding. Moreover, vitamin D deficiency further increased alcohol-induced upregulation of HO-1. These results suggest that vitamin D deficiency aggravates alcohol-induced hepatic oxidative stress possibly through promoting hepatic NAPDH oxidases and probably through regulating hepatic antioxidant enzyme genes.

In addition to oxidative stress, inflammation plays an important role in the progression of ALD [37, 38]. Several reports showed that inflammatory cytokines were significantly elevated in the patients with ALD [39, 40]. The proinflammatory cytokines, such as TNF-*α* and IL-1*β*, and chemokines, such as KC and MCP-1, have been widely considered to be the important contributors in the development of alcohol-induced liver disease [41, 42]. Indeed, vitamin D has well-established anti-inflammatory property [43, 44]. The present study showed that vitamin D deficiency elevated the alcohol-induced increase of hepatic tnf-*α*, il-1*β*, kc, and mcp-1 mRNA expressions. These results suggest that vitamin D deficiency promotes alcohol-induced inflammation. The potential mechanisms of vitamin D in regulating inflammatory responses are complex [44]. It was well known that oxidative stress stimulates the release of inflammatory cytokines and amplifies inflammatory response to cause further liver damage [45, 46]. Thus, additional study is necessary to explore the mechanism how vitamin D deficiency aggravates proinflammatory effects during chronic alcohol-induced liver injury.

Apoptosis is a highly recognized feature of ALD [47]. Several studies have observed hepatocyte apoptosis in alcohol-induced liver injury [48, 49]. An early study demonstrated that long-term vitamin D deficiency induced liver apoptosis [50]. Accordingly, the present study showed a number of apoptotic hepatocytes in alcohol-fed mice. Interestingly, alcohol-induced hepatocyte apoptosis was aggravated when vitamin D in feed was depleted. These results suggest that vitamin D deficiency promotes alcohol-induced hepatocyte apoptosis during chronic alcohol-induced liver injury. It is established that the release of inflammatory cytokines leads to hepatocyte apoptosis, as well as ROS accumulation and oxidative stress [51, 52]. In the present study, the mechanism of vitamin D deficiency that mediated proapoptosis during chronic alcohol-induced liver injury should be explored in further study.

In addition, our data also provide evidence that alcohol feeding elevated hepatic and serum TG content and resulted in an obvious hepatic lipid accumulation, which is consistent with previous studies [53]. Of note, in the present study, vitamin D deficiency had a little effect on alcohol-induced elevation of both hepatic triglyceride content and lipid accumulation. Indeed, there remains with debate concerning the role of vitamin D on lipid metabolism [54–57]. Of interest, our previous study found that vitamin D deficiency alleviates acute alcohol-induced hepatic lipid accumulation [58]. These results suggest that the effects of vitamin D deficiency on alcohol-induced hepatic lipid accumulation might correlate with the mode and time of alcohol exposure. The results in our investigation suggest that vitamin D deficiency exacerbated chronic alcohol-induced liver injury independent of hepatic TG metabolism.

In summary, the present study investigated the effects of vitamin D deficiency on chronic alcohol-induced liver injury in mice. Our results showed that vitamin D deficiency exacerbated chronic alcohol-induced liver injury, which might be associated with aggravating hepatic oxidative stress and inflammation. The present study demonstrates for the first time that vitamin D deficiency aggravates alcohol-evoked hepatic oxidative stress through regulating hepatic oxidant and antioxidant enzymes.

## Figures and Tables

**Table 1:** Oligonucleotide sequence of primers for real-time RT-PCR.

Genes	Sequences	Size (bp)
*18S*	Forward: 5′-GTAACCCGTTGAACCCCATT-3′ Reverse: 5′-CCATCCAATCGGTAGTAGCG-3′	151
*tnf-α*	Forward: 5′-CCCTCCTGGCCAACGGCATG-3′ Reverse: 5′-TCGGGGCAGCCTTGTCCCTT-3′	109
*il-1β*	Forward: 5′-GCCTCGTGCTGTCGGACCCATAT-3′ Reverse: 5′-TCCTTTGAGGCCCAAGGCCACA-3′	143
*il-6*	Forward: 5′-CTGCAAGAGACTTCCATCCAG-3′ Reverse: 5′-AGTGGTATAGACAGGTCTGTTGG-3′	131
*tgf-β*	Forward: 5′-CGGGAAGCAGTGCCCGAACC-3′ Reverse: 5′-GGGGGTCAGCAGCCGGTTAC-3′	147
*mcp1*	Forward: 5′-GGCTGGAGAGCTACAAGAGG-3′ Reverse: 5′-GGTCAGCACAGACCTCTCTC-3′	93
kc	Forward: 5′-ACTCAAGAATGGTCGCGAGG-3′ Reverse: 5′-GTGCCATCAGAGCAGTCTGT-3′	123
Crp	Forward: 5′-ATGGAGAAGCTACTCTGGTGC-3′ Reverse: 5′-ACACACAGTAAAGGTGTTCAGTG-3′	165
*mip2*	Forward: 5′-GCTGTCCCTCAACGGAAGAA-3′ Reverse: 5′-CGAGGCACATCAGGTACGAT-3′	175
*inos*	Forward: 5′-GCTCGCTTTGCCACGGACGA-3′ Reverse: 5′-AAGGCAGCGGGCACATGCAA-3′	146
*p47phox*	Forward: 5′-CCAGGGCACTCTCACTGAATA-3′ Reverse: 5′-ATCAGGCCGCACTTTGAAGAA-3′	100
*gp91phox*	Forward: 5′-GGGAACTGGGCTGTGAATGA-3′ Reverse: 5′-CAGTGCTGACCCAAGGAGTT-3′	147
*nox4*	Forward: 5′-CCAAATGTTGGGCGATTGTGT-3′ Reverse: 5′-TCCTGCTAGGGACCTTCTGT-3′	133
*gshpx*	Forward: 5′-GGTGGTGCTCGGTTTCCCGT-3′ Reverse: 5′-AATTGGGCTCGAACCCGCCAC-3′	113
*sod1*	Forward: 5′-GCGATGAAAGCGGTGTGCGTG-3′ Reverse: 5′-TGGACGTGGAACCCATGCTGG-3′	143
*gshrd*	Forward: 5′-GGGATGCCTATGTGAGCCGCC-3′ Reverse: 5′-TGACTTCCACCGTGGGCCGA-3′	107

**Table 2:** ADH and ALDH activities in the liver in different dietary groups.

Parameters	Control	VDD	EtOH	VDD+EtOH
ADH (U/mg protein)	6.72 ± 0.53	7.72 ± 0.21	16.27±2.12^∗∗^	16.27±1.38^∗∗^
ALDH (U/L)	11.24 ± 1.53	13.20 ± 1.45	32.36±3.24^∗∗^	29.26 ± 2.4^1∗∗^

Data are means ± SEM. *n* = 10/group. ^∗∗^*P* < 0.01 versus control group.

**Table 3:** Serum biochemical parameters (mmol/L).

Parameters	TG	CHOL	Chol-HDL	Chol-LDL	Chol-VLDL
Control	1.60 ± 0.14	3.78 ± 0.09	2.72 ± 0.07	0.44 ± 0.02	0.62 ± 0.03
EtOH	2.18 ± 0.18^∗^	3.64 ± 0.21	2.49 ± 0.13	0.44 ± 0.11	0.72 ± 0.04
VDD	1.83 ± 0.26	4.03 ± 0.33	2.62 ± 0.20	0.74 ± 0.19	0.67 ± 0.05
VDD+EtOH	2.14 ± 0.17	3.66 ± 0.21	2.47 ± 0.15	0.49 ± 0.11	0.70 ± 0.04

Data are means ± SEM. *n* = 10/group. ^∗^*P* < 0.05 versus control group.

## Data Availability

The data used to support the findings of the study on “Vitamin D deficiency aggravates hepatic oxidative stress and inflammation during chronic alcohol-induced liver injury in mice” are available from the corresponding author upon request.
